# Correlations between corneal biomechanics and specular microscopy in patient with cataract

**Published:** 2020

**Authors:** Raluca Claudia Iancu, Inna Adriana Bujor, Cătălina Iliuță, Ștefania Tudor, Emil Ungureanu, Irena Gabriela Pașca, Sînziana Istrate

**Affiliations:** * Department of Ophthalmology, University Emergency Hospital, Bucharest, Romania; ** ”Carol Davila” University of Medicine and Pharmacy, Bucharest, Romania

**Keywords:** corneal endothelial cell density, specular microscopy, corneal biomechanics, corneal hysteresis, Ocular Response Analyzer

## Abstract

This study aimed to analyze the connection between corneal biomechanics (corneal hysteresis, CH) and endothelial cell density of cornea (mean endothelial cell density, MCD) in patients diagnosed with cataract.

This retrospective, observational study was performed in the Ophthalmology Clinic of the University Emergency Hospital in Bucharest. Of 60 patients (120 eyes) with cataract, who were included in this study, we analyzed the CH values obtained using with the Ocular Response Analyzer (ORA) and the MCD values obtained using the specular microscopy. The study groups comprised both men and women with ages ranging from 45 to 63 years.

Patients were divided into three study groups according to CH values. In each batch, the CH values obtained with the Ocular Response Analyzer (ORA) were correlated with age, gender and MCD, then the subgroups were compared. All the data gathered showed no correlation to be statistically significant regarding the biomechanical properties of the cornea and the corneal endothelial cell density in patients with cataract.

## Introduction

This study aimed to determine a possible relationship between the biomechanical properties of the cornea and the corneal endothelial cell density in patients with cataract.

Cataract is a clouding of the lens of the eye that leads to a progressive loss of vision. This is a common ophthalmological disorder that is caused by opacification of the lens and it generally develops in both eyes, but not evenly.

Numerous studies suggest that corneal hysteresis (CH), an indicator of corneal viscoelasticity, is associated with biomechanical properties of the cornea. Some studies showed that CH may be related to the biomechanical characteristics of peripapillary sclera and lamina cribrosa, as they are embryologically developed from the mesoderm and also because the collagen fibers continue with the collagen fibers from the corneal stroma [**1**-**8**].

The ORA (Ocular Response Analyzer) is a tool that determines corneal hysteresis (CH) at the indentation by a rapid jet of air. Corneal hysteresis is defined as the difference between the tension at which the cornea bends inward during an air jet applanation and the tension at which it bends out again. The two pressure values (P1 and P2) are obtained at the end of the measurement period, which lasts approximately 20 milliseconds. 

Thus, ORA can be used for the assessment of corneal biomechanics in vivo [**9**,**10**].

Corneal hysteresis (CH) represents the difference between the two pressure determinations, P1 and P2, reflecting the capacity for absorption of kinetic energy in the tissue, an indicator of corneal viscosity. The mean of the two pressure measurements (P1 and P2) determines the Goldmann correlated intraocular pressure (IOPg) [**11**-**13**].

Specular microscopy is a photographic, noncontact technique for visualizing and analyzing the shape, size and number of the population of endothelial cells, by computer-assisted analysis of endothelial morphology. The instrument projects light onto the corneal surface and captures image from the endothelial/ aqueous interface. 

Specular microscopy is a noninvasive method for analyzing the corneal endothelium and facilitates the rapid and correct diagnosis of corneal endotheliopathy affecting the structure and physiology of this corneal layer [**14**-**17**].

Although there have been several studies analyzing the relationship between eyes with cataract and the biomechanical properties of the cornea or endothelial cell density, the results are still under debate.

## Materials and methods

This retrospective, observational study was performed in the Ophthalmology Clinic of University Emergency Hospital in Bucharest, during the year 2019.

All patients involved in the study were informed about the use of personal data, and the study was organized in accordance with the ethical principles stated in the Declaration of Helsinki, developed by the WMA (World Medical Association) [**18**].

60 patients were included in this study, patients known with changes of transparency of the lens in any of its layers, of at least 3+, pathology for which they underwent surgery. All subjects included in the study were Caucasian, aged between 45 and 63 years, with an equal gender distribution.

Data was gathered from medical records, including a complete patient history, as well as visual acuity with or without correction, refraction, biomicroscopic examination of the anterior segment and posterior pole, keratometry, pachymetry, specular microscopy, tonometry with the help of Goldman applanation and Ocular Response Analyzer (ORA).

Another inclusion criterion was a normal value of intraocular pressure (12–22 mmHg).

The exclusion criteria from the study were the following: positive history for the administration of topical eye medications and/ or for rigid or soft contact lenses use, glaucoma or glaucoma suspect, inflammation and/ or ocular infection in the background, dystrophies/ corneal pathology (Fuchs endothelial dystrophy, keratoconus, pellucid marginal corneal degeneration, bullous keratopathy) and last but not least, without any other ocular surgery procedures performed in medical history, as for example refractive surgery or laser ablation of the cornea and lens, corneal cross-linking (CXL) and intrastromal corneal ring segment (ICRS).

Paraclinical investigations were performed using the equipment of the Ophthalmology Clinic in the University Emergency Hospital, Bucharest.

Images reflecting endothelial cell density were captured using a specular microscope (Topcon, SP-3000P). Three consecutive measurements were made with a specular reflex light, a correct centering of the fixation target, with a calibration and a favorable image resolution. The measurement technique used was the center to center method. 45-50 corneal endothelial cells were counted and cell density (CD) was calculated by automatic analysis. The average of the three CD values was considered.

In vivo data of corneal biomechanics was obtained using the Ocular Response Analyzer (Reichert Ocular Response Analyzer G3 AutoTonometer, evaluation software v.1.01). The correct positioning of the patient was verified throughout the examination and three consecutive measurements (Single Measure) were performed. The average of recordings with a score higher than 7 (Waveform Score) was taken into consideration.

Goldmann applanation tonometry (GAT) was performed for IOP measurements.

This study included 60 patients, aged between 45 and 63 years, females and males, with cataract. We analyzed and compared the following parameters: corneal hysteresis (CH) and mean endothelial cell density (MCD) in both eyes.

Patients have been divided into three distinct study groups, according to the value of corneal hysteresis: batch no. 1 CH at RE = 8.33 ± 0.29 mm Hg and LE CH = 8.48 ± 0.26 mmHg, batch no 2. CH at RE = 9.33 ± 0.275 mmHg and CH at LE = 9.46 ± 0.275, batch no. 3 CH at RE = 10.62 ± 0.345 and LE CH = 10.55 ± 0.266.

SPSS statistics package 20 was used for statistical analysis.

Pearson’s correlation coefficient is the test statistics used to measure the statistical relationship, or association, between CCT (central corneal thickness), mean corneal endothelial cell density and the properties of corneal biomechanics.

For statistical significance we have considered P values ≤ 0.05.

## Results

60 patients aged between 45 and 63 years, equal in proportion, women and men, were included in the study group.

Three groups were realized according to corneal hysteresis (CH) values. The data in **Table 1** represent the description of the parameters analyzed in the study.

**Table 1:** Parameters analyzed

Parameters	RE			LE		
	Mean ±SD	AM	Min-Max	Mean ±SD	AM	Min-Max
CH Batch 1	8.33 ± 0.29	8.2	8-8.9	8.48 ± 0.26	8.5	8-8.9
MCD Batch 1	2199 ± 327.18	2106.5	1814-2805	2232.5 ± 349.34	2078	1800-2872
CH Batch 2	9.33 ± 0.275	9.3	9-9.9	9.46 ± 0.275	9.5	9-9.9
MCD Batch 2	2077.2 ± 328.84	1991.5	1668-2688	2093.3 ± 345.93	2002.5	1612-2805
CH Batch 3	10.62 ± 0.345	10.7	10.1-11.2	10.55 ± 0.266	10.5	10.1-11
MCD Batch 3	2110.25 ± 327.47	2007.5	1707-2812	2141.6 ± 319.05	2101	1602-2713

**Fig. 1:**
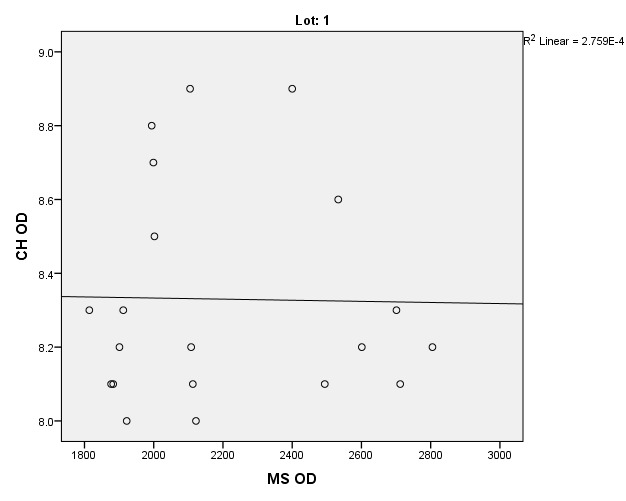
Correlations between CH RE and MCD RE in batch 1

**Fig. 2:**
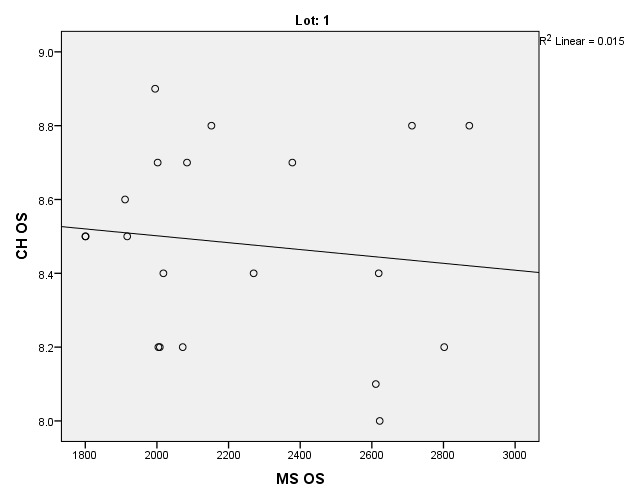
Correlations between CH LE and MCD LE in batch 1

**Fig. 3:**
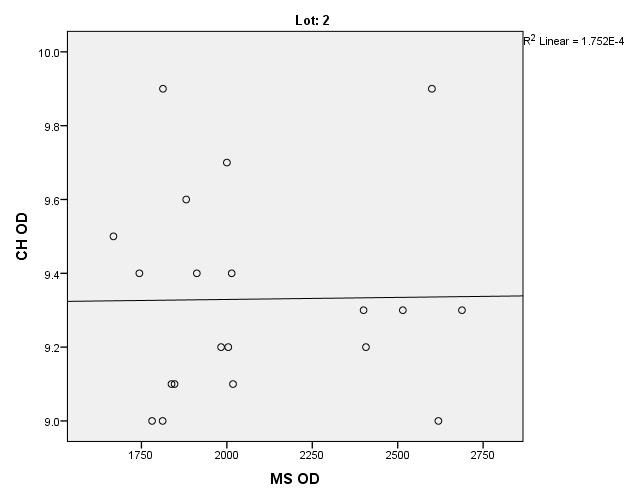
Correlations between CH RE and MCD RE in batch 2

**Fig. 4:**
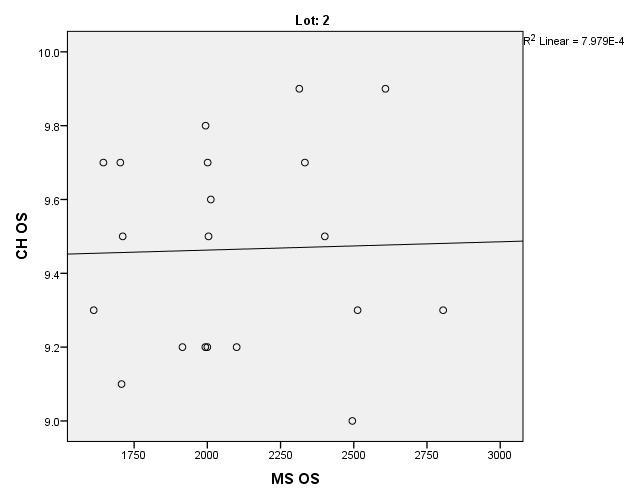
Correlations between CH LE and MCD LE in batch 2

**Fig. 5:**
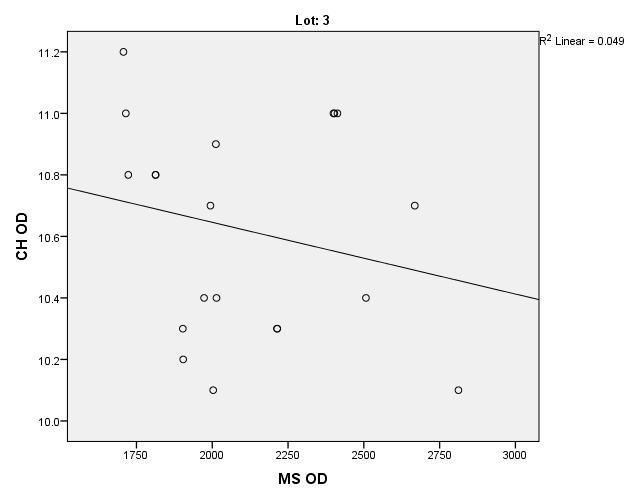
Correlations between CH RE and MCD RE in batch 3

**Fig. 6:**
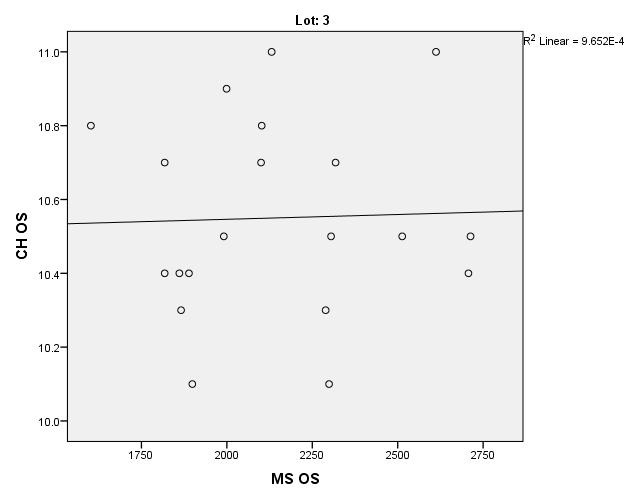
Correlations between CH LE and MCD LE in batch 3

There were correlations between corneal hysteresis and endothelial cell density (MCD) in each batch. Most variables had a non-parametric distribution according to the Shapiro-Wilk test (p <0.05) (**Tables 2**-**4**, **Fig. 1**-**6**).

**Table 2:** Correlation between corneal hysteresis and endothelial cell counts in patients in group 1

Correlation	p*
CH RE (p=0.005**) x MCD RE (p=0.011**)	0.972, R=0.008
CH LE (p=0.284**) x MCD LE (p=0.019**)	0.633, R= -0.114
*Shapiro-Wilk Test, **Spearman’s rho Correlation Coefficient	

**Table 3:** Correlation between corneal hysteresis and endothelial cell counts in patients in group 2

Correlation	p*
CH RE (p=0.062**) x MCD RE (p=0.007**)	0.962, R= -0.011
CH LE (p=0.192**) x MCD LE (p=0.200**)	0.906, R=0.028***
*Shapiro-Wilk Test, **Spearman’s rho Correlation Coefficient, ***Pearson Correlation Coefficient	

**Table 4:** Correlation between corneal hysteresis and endothelial cell counts in patients in group 3

Correlation	p*
CH RE (p=0.094**) x MCD RE (p=0.108**)	0.350, R= -0.221
CH LE (p=0.368**) x MCD LE (p=0.265**)	0.897, R=0.031***
*Shapiro-Wilk Test, **Pearson Correlation Coefficient	

According to the correlations established using Spearman’s rho coefficients, the association between corneal hysteresis and endothelial cell density was not statistically significant in the right or left eye for patients in group 1, 2 or 3.

The data in **Tables 5**-**7** compare the groups together by analyzing corneal hysteresis and endothelial cell density from patients in group 1, group 2 and respectively 3. The variables were tested for distribution according to the Shapiro-Wilk test (**Fig. 7**-**18**).

**Table 5:** Correlations between corneal hysteresis and endothelial cell density in patients in batch 1 vs. batch 2

Correlation	p*
CH RE L1(p=0.005**) x MCD RE L2 (p=0.007**)	0.525, R= -0.151
CH LE L1 (p=0.284**) x MCD LE L2 (p=0.200**)	0.626, R= -0.116***
CH RE L2 (p=0.062**) x MCD RE L1 (p=0.011**)	0.052, R= 0.441
CH LE L2 (p=0.192**) x MCD LE L1 (p=0.019**)	0.056, R= -0.434
*Shapiro-Wilk Test, **Spearman’s rho Correlation Coefficient, ***Pearson Correlation Coefficient	

**Table 6:** Correlations between corneal hysteresis and endothelial cell density in patients in batch 1 vs. batch 3

Correlation	p*
CH RE L1 (p=0.005**) x MCD RE L3 (p=0.108**)	0.636, R= -0.113
CH LE L1 (p=0.284**) x MCD LE L3 (p=0.265**)	0.069, R= 0.415***
CH RE L3 (p=0.094**) x MCD RE L1 (p=0.011**)	0.684, R= 0.097
CH LE L3 (p=0.368**) x MCD LE L1 (p=0.019**)	0.697, R= 0.093
*Shapiro-Wilk Test, **Spearman’s rho Correlation Coefficient, ***Pearson Correlation Coefficient	

**Table 7:** Correlations between corneal hysteresis and endothelial cell density in patients in batch 2 vs. batch 3

Correlation	p*
CH RE L2(p=0.062**) x MCD RE L3 (p=0.108**)	0.102, R= -0.376***
CH LE L2 (p=0.192**) x MCD LE L3 (p=0.265**)	0.018, R=0.522***
CH RE L3 (p=0.094**) x MCD RE L2 (p=0.007**)	0.765, R= -0.071
CH LE L3 (p=0.368**) x MCD LE L2 (p=0.200**)	0.116, R= 0.363***
*Shapiro-Wilk Test, **Spearman’s rho Correlation Coefficient, ***Pearson Correlation Coefficient	

**Fig. 7:**
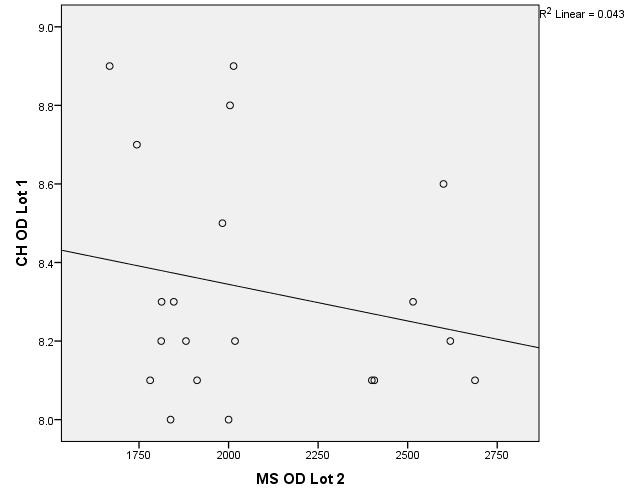
Correlation between CH RE Batch 1 and MCD RE Batch 2

**Fig. 8:**
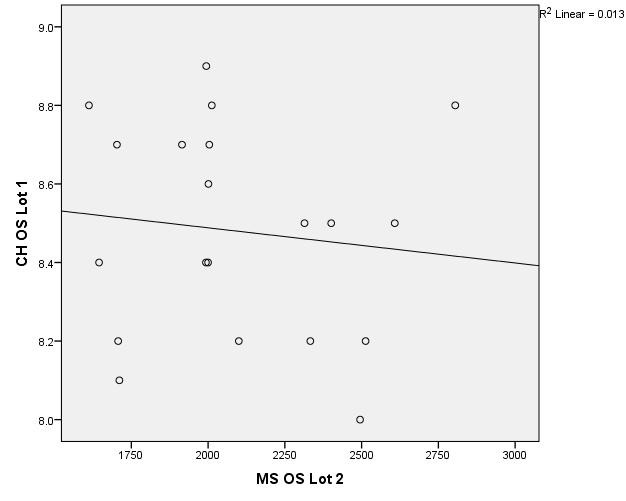
Correlations between CH LE Batch 1 and MCD LE Batch 2

**Fig. 9:**
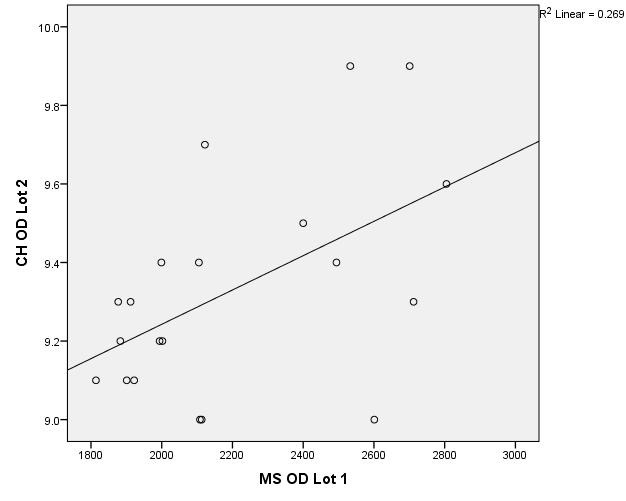
Correlation between CH RE Batch 2 and MCD RE Batch 1

**Fig. 10:**
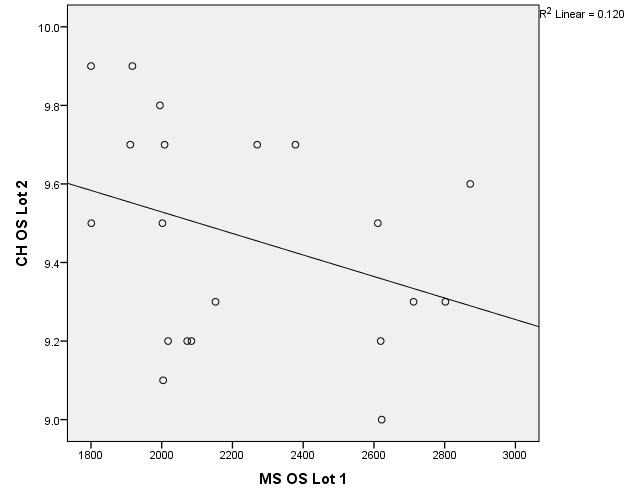
Correlation between CH LE Batch 2 and MCD LE Batch 1

**Fig. 11:**
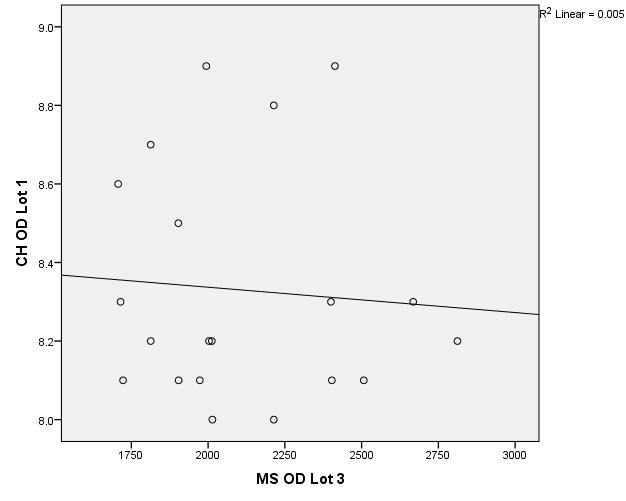
Correlation between CH RE Batch 1 and MCD RE Batch 3

**Fig. 12:**
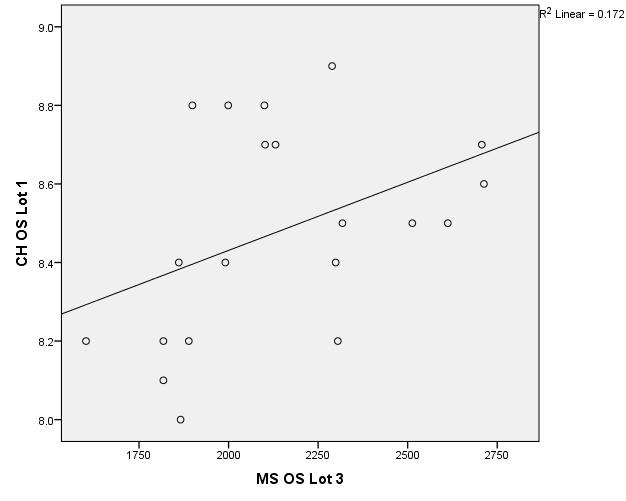
Correlations between CH LE Batch 1 and MCD LE Batch 3

**Fig. 13:**
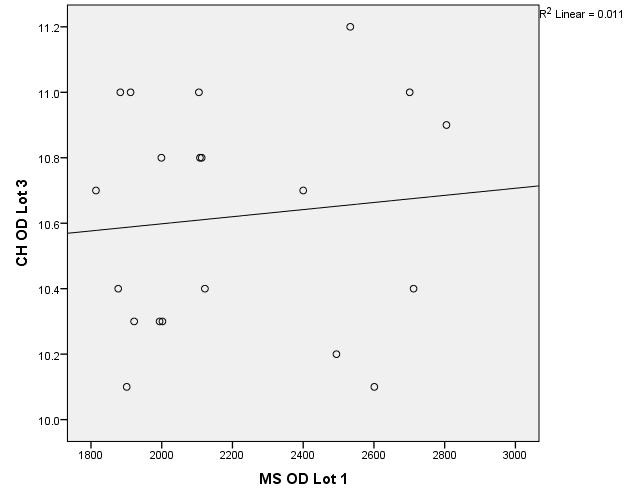
Correlation between CH RE Batch 3 and MCD RE Batch 1

**Fig. 14:**
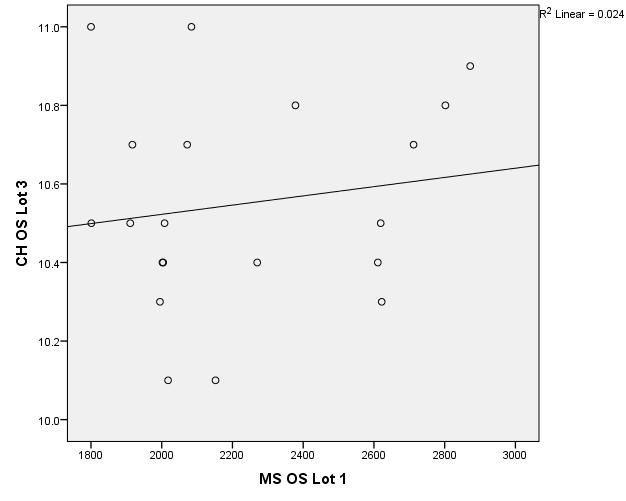
Correlation between CH LE Batch 3 and MCD LE Batch 1

**Fig. 15:**
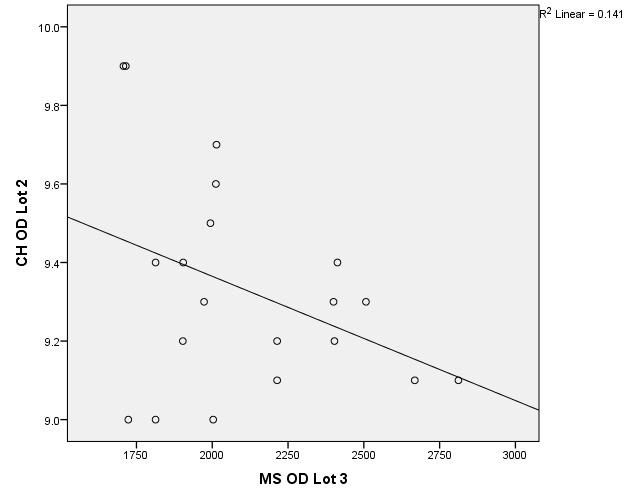
Correlation between CH RE Batch 2 and MCD RE Batch 3

**Fig. 16:**
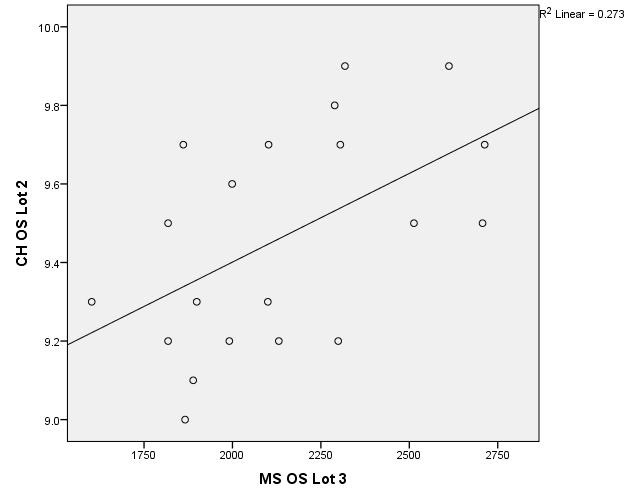
Correlations between CH LE Batch 2 and MCD LE Batch 3

**Fig. 17:**
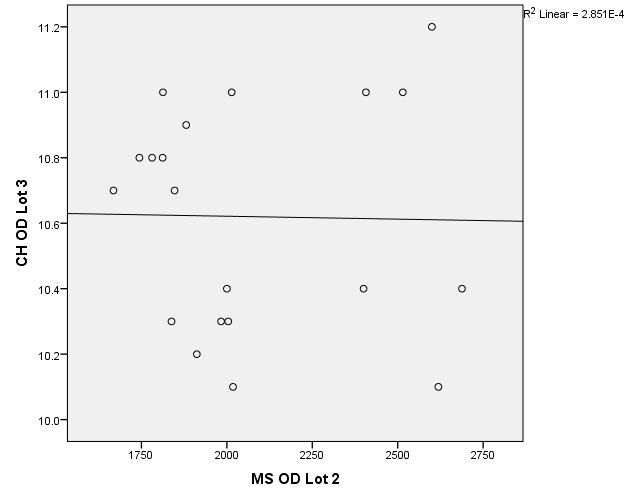
Correlation between CH RE Batch 3 and MCD RE Batch 2

**Fig. 18:**
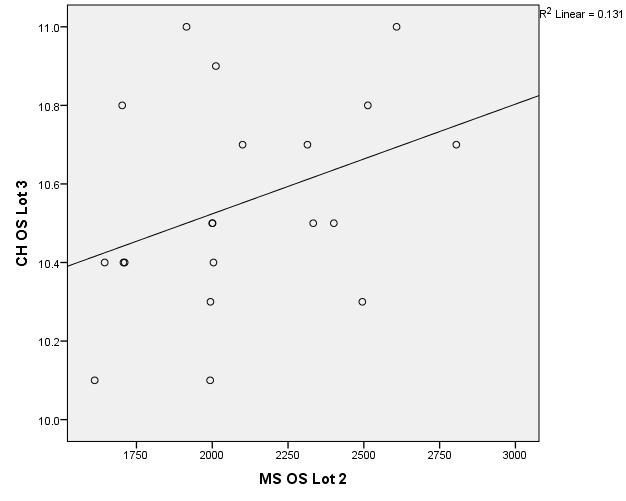
Correlation between CH LE Batch 3 and MCD LE Batch 2

According to the correlations established using Spearman’s rho/ Pearson coefficients, the associations between corneal hysteresis and endothelial cell density were not statistically significant. The exception was the correlation between the corneal hysteresis value in the left eye for group 2 of patients and the value of the number of cells in the left eye in group 3 of patients (p = 0.018, R = 0.522), thus showing a small value of CH from LE in group 2 of patients (close to 9 mmHg), which was significantly associated with a low value of the number of endothelial cells from LE in group 3 of patients and vice versa.

**Fig. 19:**
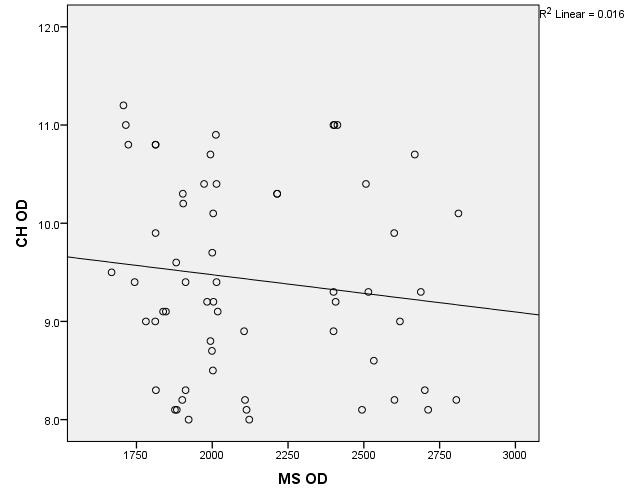
Correlations between CH RE and MCD RE throughout the study group

The data in **Table 8** and **Fig. 19**,**20** represent the correlation between corneal hysteresis and endothelial cell density in patients throughout the study group. According to the Shapiro-Wilk test (p <0.05), all variables had a non-parametric distribution.

**Table 8:** Correlation between corneal hysteresis and endothelial cell density in patients from the entire study group

Correlation	p*
CH RE (p=0.002**) x MCD RE (p<0.001**)	0.233, R= -0.156
CH LE (p=0.009**) x MCD LE (p=0.008**)	0.429, R= -0.104
*Shapiro-Wilk Test, **Spearman’s rho Correlation Coefficient	

**Fig. 20:**
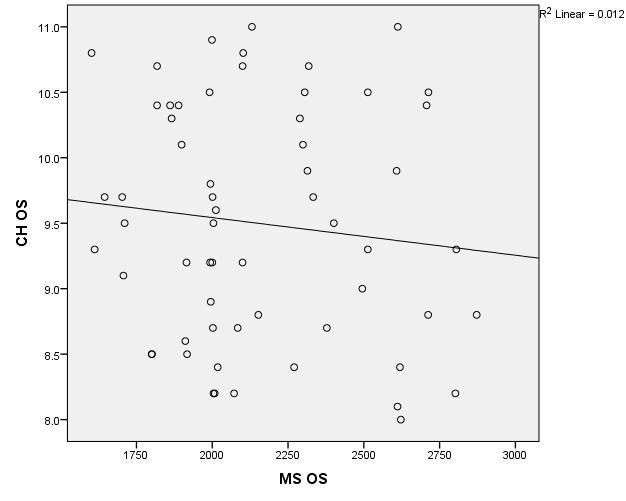
Correlation of CH LE and MCD LE across the study group

Taking into account the correlations established using Spearman’s rho coefficients, the association between corneal hysteresis and endothelial cell density was not statistically significant in the right eye (p = 0.233) or the left eye (p = 0.429) for patients in the entire study group.

**Fig. 21:**
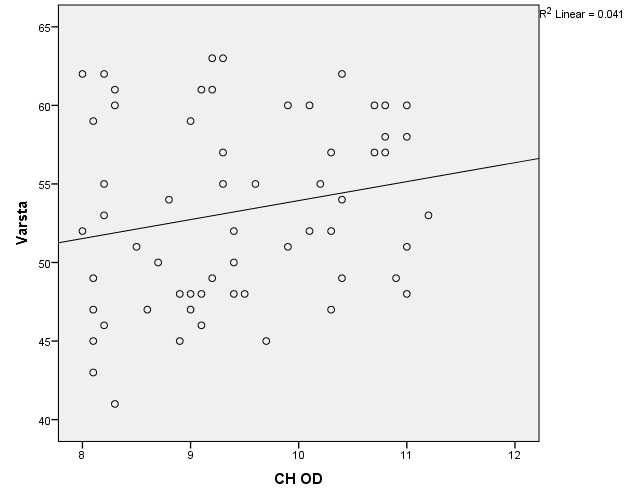
Right eye: relationship between CH and patients age

There were correlations between the age/ sex of the patients with the values of corneal hysteresis and the density of corneal endothelial cells. According to the Shapiro-Wilk test (p <0.05), the distribution of variables was non-parametric (**Table 9**-**16**, **Fig. 21**-**Fig. 28**).

**Table 9:** Right eye: relationship between CH and patients age

Correlation	p*
Age (p=0.015**) x CH RE (p=0.002**)	0.127, R=0.199
*Spearman’s rho Correlation Coefficient, **Shapiro-Wilk Test	

**Table 10:** Left eye: relationship between CH and patients age

Correlation	p*
Age (p=0.015**) x CH LE (p=0.009**)	0.084, R=0.225
*Spearman’s rho Correlation Coefficient, **Shapiro-Wilk Test	

**Table 11:** Right eye: relationship between MCD and patients age

Correlation	p*
Age (p=0.015**) x MCD RE (p<0.001**)	0.388, R= -0.114
*Spearman’s rho Correlation Coefficient, **Shapiro-Wilk Test	

**Table 12:** Left eye: relationship between MCD and patients age

Correlation	p*
Age (p=0.015**) x MCD LE (p=0.008**)	0.369, R= -0.118
*Spearman’s rho Correlation Coefficient, **Shapiro-Wilk Test	

**Table 13:** Right eye: relationship between CH and patient gender

Gender	Mean ± SD	Median Rank	p*
Female (p=0.075**)	9.39 ± 0.985	29.92	0.790
Male (p=0.021**)	9.465 ± 1.015	31.12	
*Mann-Whitney U Test, **Shapiro-Wilk Test			

**Table 14:** Left eye: relationship between CH and patient gender

Gender	Mean ± SD	Median Rank	p*
Female (p=0.026**)	9.51 ± 0.946	30.89	0.859
Male (p=0.263**)	9.482 ± 0.848	30.09	
*Mann-Whitney U Test, **Shapiro-Wilk Test			

**Table 15:** Right eye: relationship between MCD and patient gender

Gender	Mean ± SD	Median Rank	p*
Female (p=0.004**)	2197.84 ± 313.26	35.74	0.016
Male (p=0.001**)	2055.66 ± 329.51	24.9	
*Mann-Whitney U Test, **Shapiro-Wilk Test			

**Table 16:** Left eye: relationship between MCD and patient gender

Gender	Mean ± SD	Median Rank	p*
Female (p=0.033**)	2208.84 ± 322.43	33.71	0.141
Male (p=0.076**)	2099.1 ± 349.84	27.07	
*Mann-Whitney U Test, **Shapiro-Wilk Test			

**Fig. 22:**
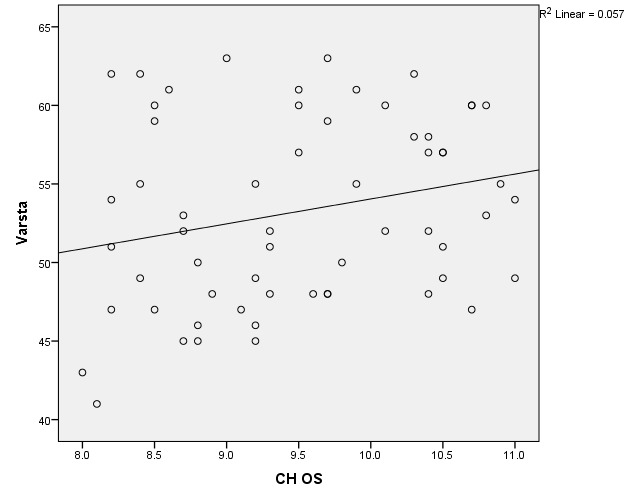
Left eye: relationship between CH and patients age

**Fig. 23:**
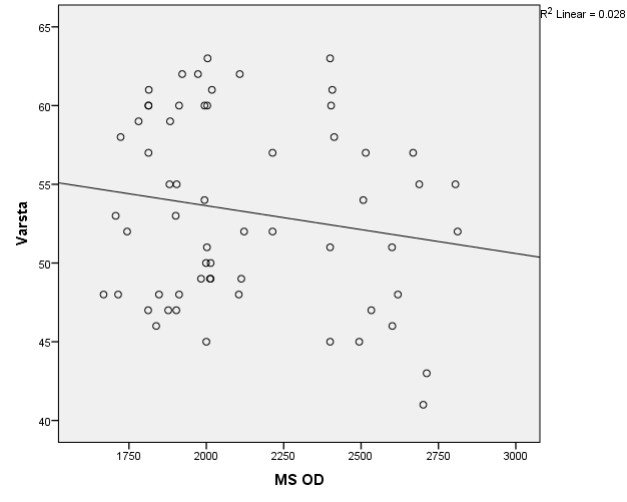
Right eye: relationship between MCD and patients age

**Fig. 24:**
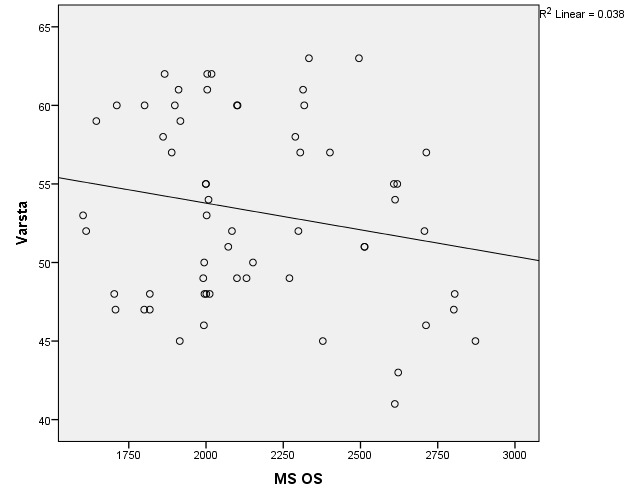
Left eye: relationship between MCD and patients age

**Fig. 25:**
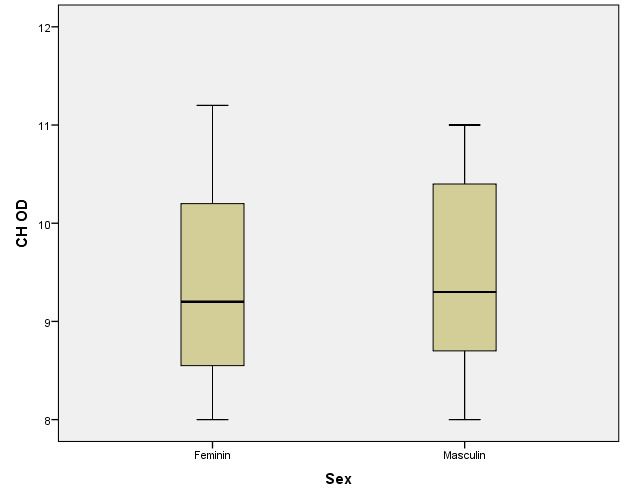
Right eye: relationship between CH and patient gender

**Fig. 26:**
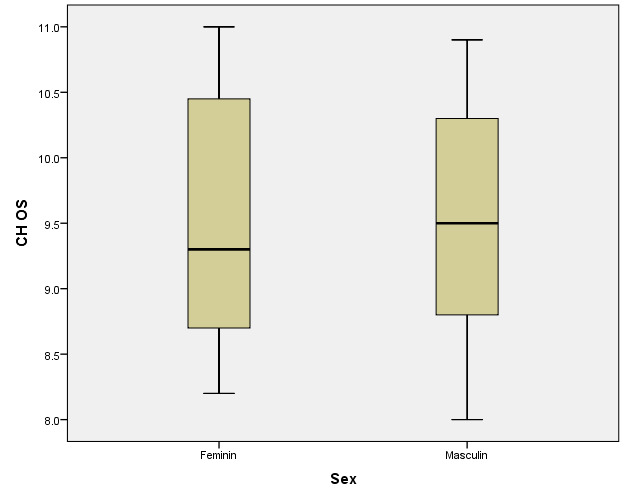
Left eye: relationship between CH and patient gender

**Fig. 27:**
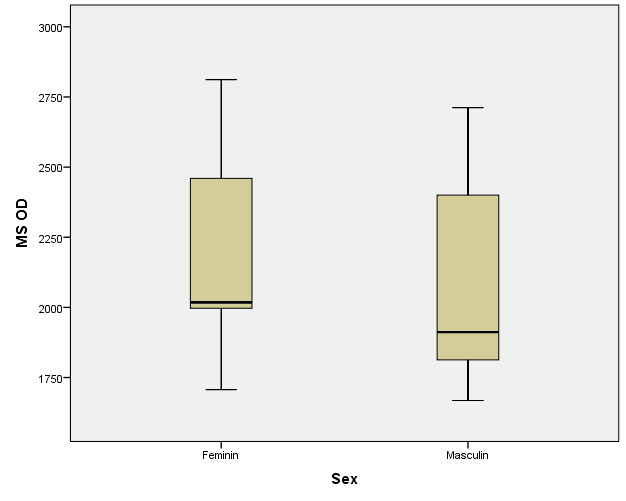
Right eye: relationship between MCD and patient gender

**Fig. 28:**
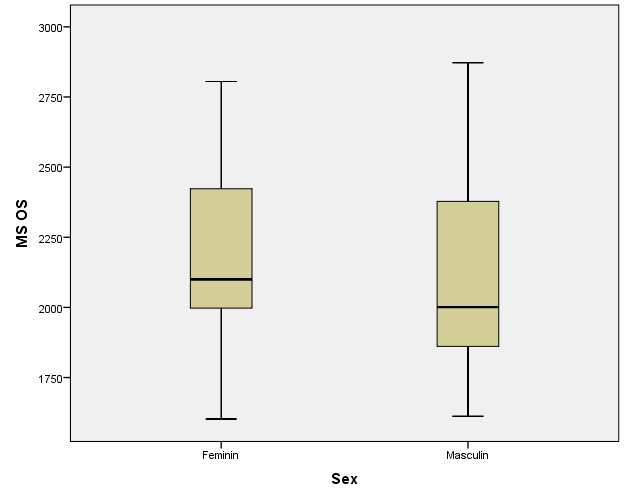
Left eye: relationship between MCD and patient gender

According to Spearman’s rho correlation coefficient, these associations were not statistically significant. According to the Mann-Whitney U test, the differences between women and men were not statistically significant for corneal hysteresis values, but the women in the study had significantly higher corneal endothelial cells (mean range = 35.74) than men (mean range = 24.9) (p=0.016).

## Discussions

Many medical studies have underlined a link between the lower values of CH and different corneal pathologies like keratoconus, corneal dystrophy, corneal edema, post LASIK status, etc. These conditions reflect the disorganization of the collagen fibers at the corneal stroma level. 

Low values of the CH have also been identified in patients with primary open-angle glaucoma and normal-tension glaucoma. Thus, they sustain the hypothesis that changes at the Lamina Cribrosa level are directly linked with the corneal biomechanical alteration. Because of this, the CH measurements are useful in the normal-tension glaucoma and early keratoconus [**19**-**22**].

Numerous studies have found a clear correlation between corneal hysteresis and CCT (central corneal thickness). The hypothesis that the corneal thickness growth is associated with viscosity growth is a consequence of the fact that CH reflects the viscoelastic properties of the cornea. 

Concerning open angle glaucoma patients, many studies have pointed out the link between the CH and the intraocular pressure (IOP). For these patients, the CH value has been significantly lower than the control group. After the IOP decrease under 27 mmHg, the CH level has been partially normalized. Another observation of the authors was that, while in normal, physiological conditions, CH modifications were not correlated with IOP, for IOP values of over 21 mmHg CH modifications being associated.

This is justified by the structural modifications of the collagen fibers that suffer a significant elongation under high IOP values, thus the difference between the P1 and P2 parameters at ORA is decreasing [**23**-**26**].

Age-related changes have shown there is a positive correlation between viscoelastic properties of the cornea and CCT (central corneal thickness). Thereby, the CH and CRF (corneal resistance factor) values are decreasing, this being linked directly with the augmentation of the corneal hydration after a certain age [**27**-**30**].

For hyperopic and for female patients, higher values for CRF have been observed. For highly myopic patients, other studies have registered lower values of the CH and CRF and higher values of IOPcc (Corneal Compensated Intraocular Pressure) and of IOPg (Goldmann Correlated IOP value) compared to the emmetropic, hyperopic patients or patients with medium or low myopia [**31**-**34**].

The recent studies (since 2015), which have analyzed the mechanical corneal properties in relation with the cell density of the corneal endothelium, have been unable to show a statistically significant correlation [**35**,**36**].

The IOP growth during the cataract surgery can justify the biomechanical properties of eye globe appearing after surgery. In cataract surgery, the IOP value in the anterior chamber can grow up to approximately 50-60 mmHg; these high pressures predispose the eye tissue to degradation [**37**-**39**].

The cataract intervention through phacoemulsification leads to important endothelial corneal damage, which is extremely significant in patients with a low number of endothelial cells. Specular microscopy records a leakage of approximately 14% (11.4% -16.6%) of corneal endothelial cells.

The thickness of the central cornea grows significantly after cataract surgery for the first seven days but after that period, up to 3 months, it decreases to a value smaller than the one pre-surgery [**40**,**41**].

In the studies undergone until present, significant differences have been observed between the biomechanical corneal values pre- and post-surgery. The CH value has decreased in the immediate post-surgically period and went back to the pre-surgically level in 1-2 weeks; no statistically significant change has been identified in the CRF values [**42**-**44**].

## Conclusions

During this study, we have analyzed the following parameters: corneal hysteresis (CH) and the mean endothelial cell density (MCD) in the right and the left eye in 60 patients, males and females, aged between 45 and 63 years, with cataract.

There were no statistically significant variations of the corneal hysteresis for the right eye and for the left eye (p = 0.204) and there were no statistically significant differences between the cell count for the right eye and the left eye (p= 0.138) in all the study groups.

According to the Pearson correlation coefficient in the study batch no. 1, the correlation between the corneal hysteresis value (CH) and the mean endothelial cell density (MCD), was not statistically significant (RE p = 0.972 and LE p = 0.633).

Like batch 1, batches 2 and 3 have shown no statistically significant relationship between the value of the CH and MCD (batch 1: RE p = 0.962 and LE p =0.906, batch 2 RE p=350 and LE p=0.897).

Applying the Pearson correlation coefficient in order to determine if there was a statistically significant association between study group no. 1 and no. 2, we found that in RE p ˃ 0.05 and in LE p ˃ 0.05, therefore the association was not statistically significant.

There was no statistically significant association between study group no. 1 and no. 3 (RE p ˃ 0.05 and LE p ˃ 0.05), according to Pearson correlation coefficient.

However, we found a statistically significant correlation (p = 0.018, R = 0.522) between the CH of the LE measured in batch no. 2. and the MCD in LE in the study group no. 3.

According to the Pearson correlation, we did not find any statistically significant associations (RE p = 0.233 and LE p = 0.429) between the CH and the MCD in patients in groups no. 1, 2 and 3 in one place.

In conclusion, there was no statistically significant correlation between the biomechanical properties of the cornea and the endothelial cornea cell in patients with cataract.

**Acknowledgements**

The authors gratefully acknowledge Programul Operațional Capital Uman 2014–2020 for the data provided by Reichert Ocular Response Analyzer supported through POCU/91/4/8/109169 (Cod SMIS 2014+: 109169) “Diagnosticul și terapia bolilor rare sistemice cu afectare oculară – OCURARE”.
